# Supplementary material for: A New Strategy for Identifying Mechanisms of Drug-drug Interaction Using Transcriptome Analysis: Compound Kushen Injection as a Proof of Principle
Source: Sci Rep. 2019 Nov 4;9:15889. doi: 10.1038/s41598-019-52375-3 (PMC6828681; doi:10.1038/s41598-019-52375-3)
Supplement: Supplementary file 1 — Supplementary Information [file 41598_2019_52375_MOESM1_ESM.pdf]

A New Strategy for Identifying Mechanisms of Drug-drug Interaction Using Transcriptome Analysis: Compound Kushen Injection as a Proof of Principle

Running title: Identification of drug-drug interaction mechanisms from transcriptome data

Hanyuan Shen[1], Zhipeng Qu[1], Yuka Harata-Lee[1], Jian Cui[1], Thazin Nwe Aung[1], Wei Wang[2], R. Daniel Kortschak[1], and David L. Adelson[1]

[1]Zhendong Australia - China Centre for Molecular Chinese Medicine, School of Biological Sciences, University of Adelaide, Adelaide, South Australia, 5005.

[2] Zhendong Research Institute, Shanxi-Zhendong Pharmaceutical Co Ltd, Beijing, China.

*Corresponding author: David L. Adelson

Department of Molecular and Biomedical Science, School of Biological Sciences, University of Adelaide, Adelaide, South Australia, 5005.

Telephone: +61 8 8303 7555

Email: david.adelson@adelaide.edu.au

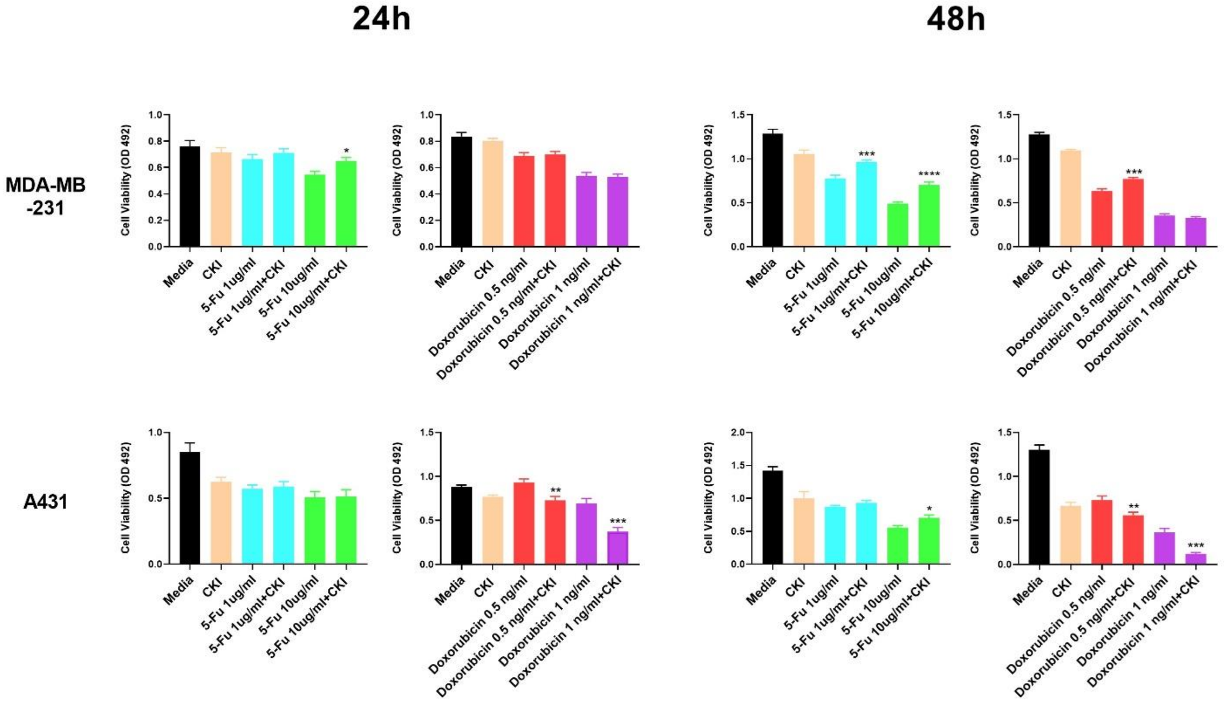

Supplementary Figure 1: Cell viability of cancer cells screened for the interaction of CKI with different chemotherapy drugs. Results are represented as mean ±SEM (N=3). Statistical analysis was performed with t-test by comparing each 'CKI + Chemotherapy Agent' to 'Chemotherapy Agent only'. (*$p<0.05$, **$p<0.01$, ***$p < 0.001$, **** $p < 0.0001$)

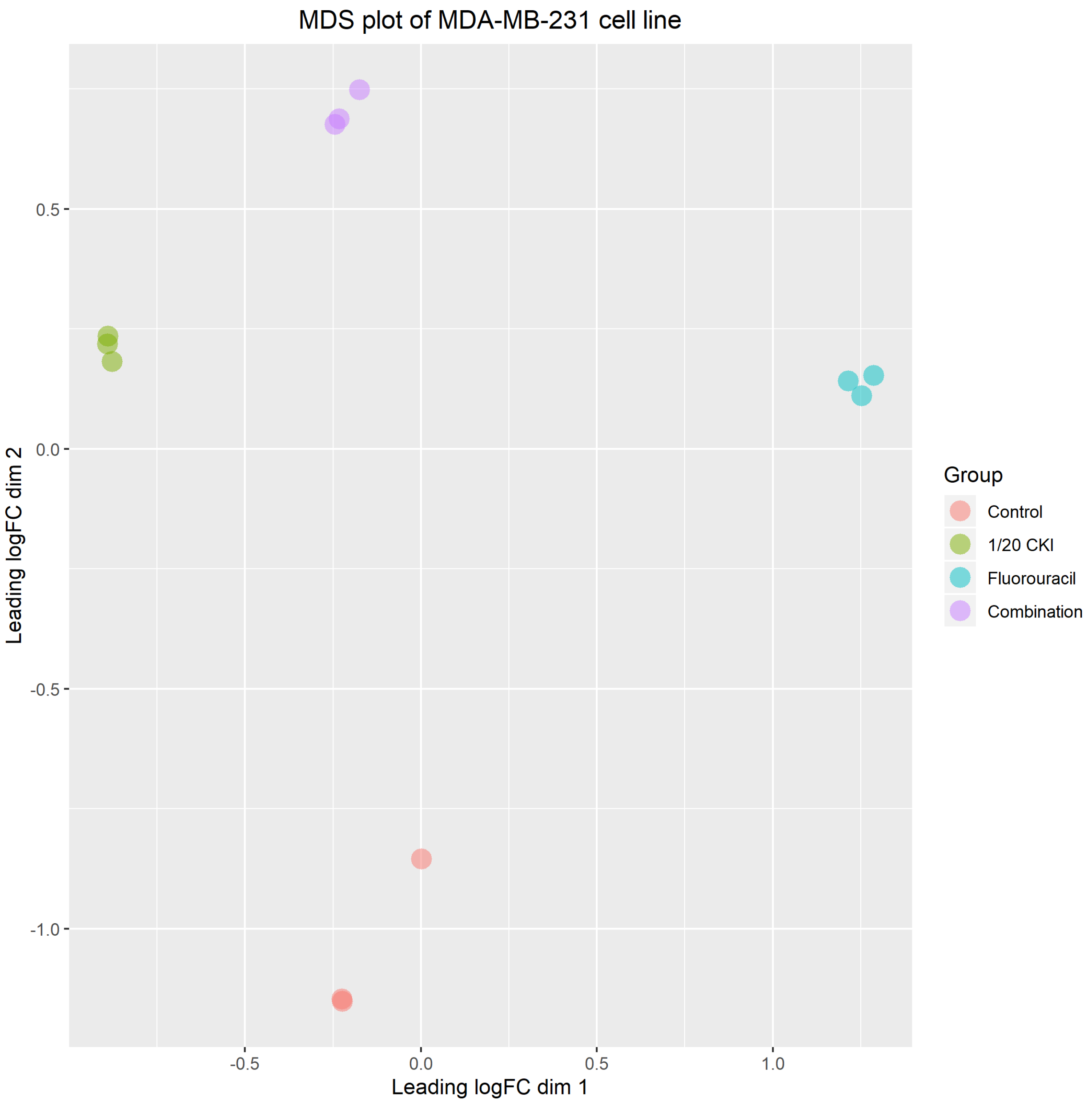

Supplementary Figure 2: Multiple dimensional scaling (MDS) plot for MDA-MB-231 samples based on expression profiles of all genes.

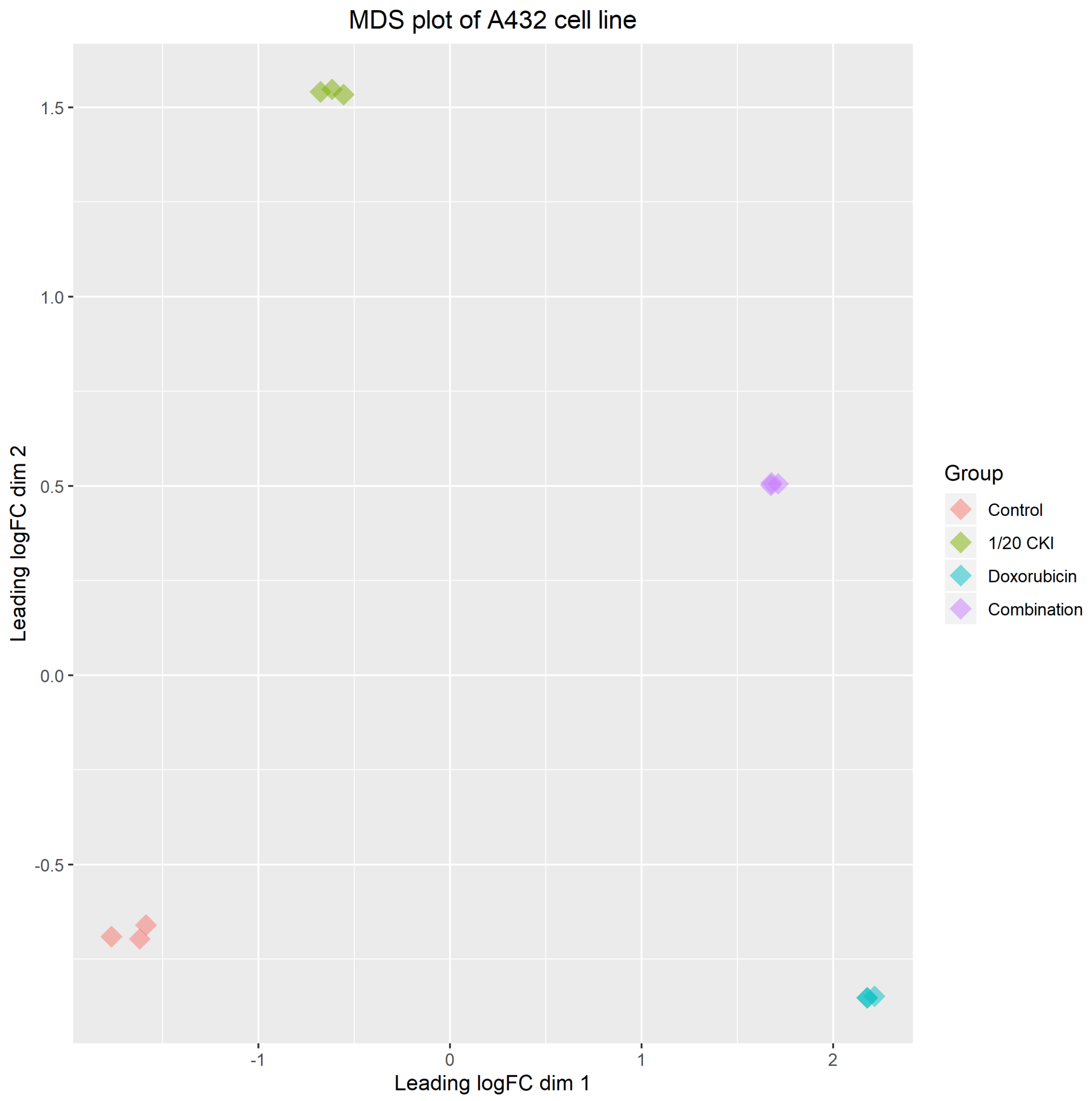

Supplementary Figure 3: Multiple dimensional scaling (MDS) plot for A431 samples based on expression profiles of all genes.

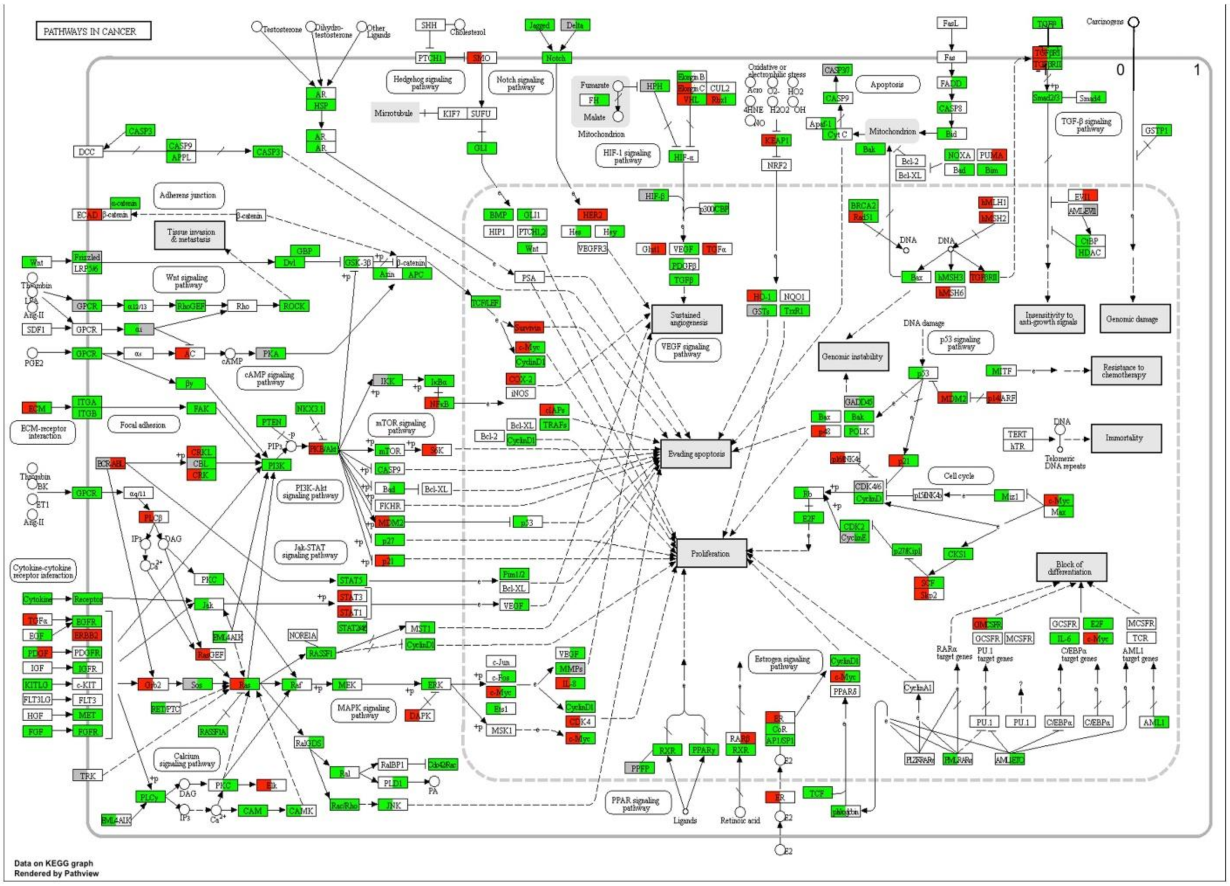

Supplementary Figure 4: Comparison of types of regulation for CKI with doxorubicin and 5-Fu in the “Pathways in cancer” pathway. Left half of the rectangle for each gene represents CKI with doxorubicin in A431 cells and the right half represents CKI with 5-Fu in MDA-MB-231 cells. Red and green colors indicate agonistic and antagonistic regulation, respectively.

Supplementary Table 1: Mapping rates for RNA-seq data.

Supplementary Table 2: List of DE genes for different comparisons.

Supplementary Table 3: Gene list for groups based on type of regulation (Group A-D) and their over-represented GO terms (count > 4 and P-value < 0.05).

Supplementary Table 4: Module-trait relationships for co-expression analysis, gene list for red module and their over-represented GO/ KEGG terms (count > 4 and P-value < 0.05).
